# Jieduquyuziyin Prescription-Treated Rat Serum Suppresses Activation of Peritoneal Macrophages in MRL/Lpr Lupus Mice by Inhibiting IRAK1 Signaling Pathway

**DOI:** 10.1155/2019/2357217

**Published:** 2019-11-03

**Authors:** Lina Ji, Xiaoli Hou, Xian Deng, Xuemin Fan, Aiwen Zhuang, Xiufeng Zhang, Rongqun Li

**Affiliations:** ^1^The First College of Clinical Medicine, Zhejiang Chinese Medical University, Hangzhou 310053, Zhejiang, China; ^2^Academy of Chinese Medical Science, Zhejiang Chinese Medical University, Hangzhou 310053, Zhejiang, China; ^3^School of Basic Medical Sciences, Zhejiang Chinese Medical University, Hangzhou 310053, Zhejiang, China; ^4^Institute of TCM Literature and Information, Zhejiang Academy of Traditional Chinese Medicine, Hangzhou 310012, Zhejiang, China; ^5^Department of Pharmacy, People's Hospital of Rizhao, Rizhao 222000, Shandong, China

## Abstract

Systemic lupus erythematosus (SLE) is a chronic autoimmune disease, and Jieduquyuziyin prescription (JP) is a traditional Chinese medicine (TCM) formula that has been testified to be effective for SLE treatment as an approved hospital prescription for many years in China. However, its mechanism of action in the treatment of this disease is largely unknown. The purpose of this study was to determine whether JP-treated rat serum can inhibit the activation of peritoneal macrophages in MRL/lpr mice by downregulating the IRAK1 signaling pathway, thereby achieving the effect of improving SLE. The JP-treated rat serum was prepared, and the peritoneal macrophages of MRL/lpr lupus mice were isolated in vitro, and the effect of JP on cell viability was detected by the CCK8 method. After LPS induction and shRNA lentiviral transfection, the effect of JP on the expression of IRAK1 in cells was detected by immunofluorescence staining. The content of TNF-*α* and IL-6 in the cell supernatant was determined by ELISA. The expression of IRAK1, NF-*κ*B, TNF-*α*, and IL-6 mRNA was detected by RT-PCR, and the expression levels of IRAK1, p-IRAK1, TRAF6, IKB*α*, p-IKB*α*, IKK + IKK, NF-*κ*B, and p-NF-*κ*B proteins was detected by western blot method. We investigated the role of JP in peritoneal macrophages of the MRL/lpr mouse and identified the possible mechanisms of action. The results showed that JP could reduce the phosphorylation of IRAK1 and its downstream proteins induced by LPS and inhibit the expression of inflammatory cytokines, including TNF-*α* and IL-6. In addition, after the transfection of cells with shRNA lentiviral, the results of JP tended to be consistent. In conclusion, JP may inhibit the activation of peritoneal macrophages in MRL/lpr mice by downregulating the IRAK1-NF-*κ*B signaling pathway, and IRAK1 may be a potential target for JP treatment of SLE.

## 1. Introduction

Systemic lupus erythematosus (SLE) is a refractory autoimmune disease involving multiple organs and systems, and its clinical manifestations are complex. SLE is characterized by the production of plenty of autoantibodies and immune complex deposition, which is likely to cause systemic or local tissue damage and organ damage [1, 2]. Glucocorticoids are still very effective treatments for SLE, but their long-term use will cause certain metabolic disorders, delay in tissue healing, and other adverse reactions [3, 4]. Therefore, to find the new drug is still crucial for the treatment of SLE.

Traditional Chinese medicine has a great potential for the treatment of autoimmune diseases. Jieduquyuziyin prescription (JP) is an empirical formula for treating SLE [5, 6], which consists of 10 kinds of herbs and has been widely used in Zhejiang Provincial Hospital of Traditional Chinese Medicine for decades. A series of biochemical experiments and clinical evidence show that it can regulate immunity, control inflammation, reduce the side effects of western medicines, and lower the incidence rate of infection [7]. The composition and therapeutic effects of JP have been investigated by many studies [8]. A research demonstrated that JP-treated rat serum could reduce GC toxicity and improve the efficacy of GC-based SLE treatment by downregulating the BAFF/BAFF-R signaling pathway [9]. For MRL/lpr lupus mice that received treatment, the pathological damage of the kidneys was significantly improved; the serum levels of IL-4 and IFN-*γ* were decreased; and the mechanism of JP on SLE was studied from the perspective of DNA methylation regulation [5]. Our previous research studied the mechanism of action for SLE induction as well as the effects of JP-treated rat serum on MeCP2 gene and protein expression and DNA methylation level in Jurkat T cells [10]. JP has been developed for the treatment of SLE, but it is still valuable to study its mechanism of action in combating the disease.

The autoimmune system is very important in the pathogenesis of SLE [11], while Toll-like receptors (TLRs) play a leading role and their abnormal expression or overactivation may lead to the onset of SLE [12]. The IRAK family is an important protein kinase involved in TLR signaling. And interleukin-1 receptor-associated kinase 1 (IRAK1) is a key signal regulator which plays a positive role in regulation of the IRAK family [13]. Hence, the inhibition of the IRAK1 signaling pathway is beneficial in reducing tissue damage mediated by the inflammatory cascade. Nuclear factor *κ*B (NF-*κ*B) is a redox-sensitive transcription factor that regulates immune and inflammatory responses. Abnormal expression of NF-*κ*B has been demonstrated in patients with SLE [14]. Activation of the NF-*κ*B pathway leads to overproduction of inflammatory cytokines such as IL-1*β*, IL-6, and TNF-*α* [15]. In addition, IRAK1 is a key regulator of the NF-*κ*B pathway and plays an important role in the pathogenesis of autoimmune diseases [16].

As an important part of the body's innate immunity, macrophages distribute widely, powerfully, and malleably. They play an important role in inflammatory response and host defense and became an important target for human immune system. Studies have shown that abnormal function of macrophages can cause imbalance of immune homeostasis and induce autoimmune diseases [17–19]. Studies have also found that macrophages played a significant role in the pathogenesis of SLE. Removal of macrophages from lupus model mice can significantly alleviate SLE nephritis and disease activity [20]. Studies suggested that there were abnormalities in immunoregulation and phagocytosis of macrophage in SLE patients, which may be involved in its pathogenesis [21]. The above studies demonstrate that macrophages are closely related to the pathogenesis of SLE. Therefore, we hypothesized that inhibition of IRAK1 activity might attenuate macrophage activity and further improved the inflammatory response of SLE by reducing the secretion of macrophage inflammatory factors.

In the known mouse models of spontaneous lupus, MRL/lpr mice develop similar pathological reactions to that of SLE patients, like renal damage as well as abnormal horizontal expression of cytokines IL-2, IL-4, IL-17, and TNF-*α*. The purpose of the current study was to analyze the effect of JP-treated rat serum on the IRAK1 signaling pathway in SLE at the cellular level by in vitro molecular experiments. We found that JP could inhibit the activation of peritoneal macrophages in MRL/lpr lupus mice by regulating the IRAK1-NF-*κ*B signaling pathway to achieve a certain therapeutic effect on SLE.

## 2. Materials and Methods

### 2.1. Preparation of JP

JP consisting of 10 herbs (Table 1) was purchased from Zhejiang Chinese Medical University Medical pieces, Ltd. (Hangzhou, China). Following ten herbs were mixed in a ratio of 5 : 4 : 4 : 5 : 4 : 5 : 4 : 3 : 3 : 2 to make 2 g/mL aqueous extract for further use: *Rehmannia glutinosa* (Libosch.), *Trionyx sinensis* Wiegmann, *Artemisia annua* (L.), *Hedyotis diffusa* (Willd.), *Paeonia veitchii* Lynch, *Centella asiatica* (L.), *Paeonia suffruticosa* (Andr.), *Citrus medica* (L.), *Cimicifuga foetida* (L.), and *Glycyrrhiza uralensis* (Fisch.). Furthermore, the extracts were mixed and concentrated to 2 g crude drug per mL for further use.

According the RRLC-QqQ/MS method used [8, 22], the content of each component in JP (*μ*g/mL) was as follows: catapol, paeoniflorin, ferulic acid, liquiritin, rutin, hesperidin, quercetin, asiaticoside, and glycyrrhizic acid. The contents were used for the analysis and quality control of JP.

### 2.2. Animals

Thirty male Sprague Dawley (SD) rats, 6–8 weeks old, 200 ± 20 g body weight, were purchased from Xipuerkekai Experimental Animal Co., Ltd. (Shanghai, China). The experimental animal production license number is SCXK (Shanghai) 2013–0016. These animals were all kept in the barrier environment of the Animal Experimental Center of Zhejiang Chinese Medical University (room temperature 20°C, relative humidity 40%–60%, and daylight 12 h), isolated in a fully enclosed clean state, free to drinking and feeding. The experimental animal license number is SYXK (Zhejiang) 2013-0184.

Twenty female MRL/lpr lupus mice, 12–16 weeks old, body weight 45 ± 2.5 g body weight, were purchased from Shanghai Slac Laboratory Animal Co., Ltd. (Shanghai, China). The experimental animal production license number is SCXK (Shanghai) 2017-0005. They were all kept in the barrier environment of the Animal Experiment Center of Zhejiang Chinese Medical University (room temperature 20°C, relative humidity 40%–60%, and daylight 12 h), isolated in normal closed specific pathogen-free (SPF) state, free to drinking and feeding. The experimental animal license number is SYXK (Zhejiang) 2018-0012.

All studies were approved by the Committee on the Ethics of Animal Experiments of Zhejiang Chinese Medical University.

### 2.3. Preparation and Culture of Mouse Macrophages

Murine peritoneal macrophages were harvested with 8 mL of cold FBS-free DMEM (Gibco, USA) supplemented with L-glutamine (4.5 mg/mL) and glucose (4.5 mg/mL) from MRL/lpr mice. The cells were washed twice with PBS and suspended in culture medium (DMEM supplemented with 10% fetal bovine serum (FBS, Gimini), penicillin (100 units/mL, Gibco), and streptomycin (100 mg/mL, Gibco)). Cells were incubated for 4 h at 37°C in a 5% CO_2_ atmosphere and were washed with PBS to remove nonadherent cells. Then, the adherent cells were used as peritoneal macrophages and were cultured with fresh media.

### 2.4. Preparation of JP-Treated Serum

Thirty SD rats were randomly divided into control group, prednisone acetate group, and JP group. According to the equivalent dose of clinical adults, the maximum daily JP was calculated based on the body weight and administered to the rats by intragastric administration with 2 mL/100 g each time in the JP group [10]. The rats in the control group were gavaged the same amount of distilled water as the JP group and the prednisone acetate (Xianju, Zhejiang, China) group. All rats were fed twice a day, once in the morning and once afternoon, and gavaged for 5 consecutive days. The blood samples were collected separately from the abdominal aorta and allowed to stand at room temperature for 1 h. After being centrifuged at 3000 rpm/min at 4°C for 15 min, the serum of both groups was isolated and stored at −80°C until required [23].

The analysis and quality control of JP-treated rat serum were identified by the liquid chromatography-mass spectrometry (LC-MS). Two of the major constituents including paeoniflorin and ferulic acid were identified in our previous study [10].

### 2.5. Cell Proliferation Assay

According to the instructions, the proliferation level of peritoneal macrophages from MRL/lpr mice was tested by Cell Counting Kit-8 kit (CCK-8, Bryotime Biotechnology, Shanghai, China). The cell concentration was adjusted to 10^5^/ml, 100 *μ*l cells were added to 96-wells plates, and the supernatant was removed after 4 hours. A volume fraction of JP-treated rat serum medium was applied to the cells, and the culture was continued for 24 hours. 10 *μ*l of CCK8 solution was added to each well with incubation for 1 h in the dark, and the optimal concentration of JP-treated rat serum was measured at 450 nm (Thermo Scientific, USA).

### 2.6. Immunofluorescence Staining

MRL/lpr mice peritoneal macrophages were fixed with 4% paraformaldehyde for 30 min at room temperature, permeabilized with 0.3% Triton X-100 for 30 min, blocked with 3% bovine serum albumin for 30 min at room temperature, incubated with IRAK1 primary antibodies (1 : 100, Abcam, ab238, UK) overnight at 4°C, and subsequently incubated with secondary antibodies goat anti-rabbit IgG (1 : 1000, Abcam, ab6717, UK) at 37°C for 30 min. Nuclei were stained with DAPI for 5 min, and cells were visualized with the fluorescence microscope (Zeiss LSM 880, Germany).

### 2.7. Cell Transfection

Construction of the IRAK1 knockdown short hairpin RNA (shRNA) lentiviral vector and plasmid extraction were performed by Gemanditech Co., Ltd. (Shanghai, China). The lentiviral interference vector was pGMLVSC5 RNAi, including a targeting IRAK1 lentiviral vector (sequence 5′-TACCGAGCAGTCATGAGAAAT-3′) and one negative control shRNA lentiviral vector (sequence 5′-TTCTCCGAACGTGTCACGT-3′). Construction of IRAK1 overexpressing shRNA lentiviral vector and plasmid extraction was performed by Genechem Co., Ltd., (Shanghai, China). The vector GV492 was digested with BamHI/AgeI, and amplified by PCR to obtain an IRAK1 gene fragment (forward 5′- AGGTCGACTCTAGAGGATCCCGCCACCATGGCCGGGGGGCCGGGC-3′, reverse 5′-TCCTTGTAGTCCATACCTATATGATTGATGAATAATTC-3′). After the shRNA lentiviral packaging was completed, the peritoneal macrophages of MRL/lpr lupus mice were transfected. 4 hours before transfection, cells were added to a 6-well plate. Then, the cells were transfected by using 0.5 *μ*g/mL polybrene (Genechem, Shanghai, China) in DMEM culture medium with shRNA according to the manufacturer's instruction for 6 h. After transfection, the mixtures were replaced with complete culture medium. Transfection efficiency was verified at 72 h by the expression level of IRAK1 protein through western blot.

### 2.8. Enzyme-Linked Immunosorbent Assay (ELISA)

Peritoneal macrophages were grown in a 6-well plate and treated with LPS, shRNA alone or with JP-treated rat serum. The levels of TNF-*α* and IL-6 in the culture medium were detected by ELISA kits (NOVUS biologicals, SLLC, USA) according to the manufacturer's instructions.

### 2.9. Reverse Transcription-Polymerase Chain Reaction (RT-PCR)

Total RNA was extracted from cells by using RNAiso Plus (Takara, China) and then reverse transcribed to cDNA using PrimeScript® RT reagent kit (Takara, China) according to the manufacturer's procedure. The cDNA was amplified by TB Green Premix Ex Taq RT-PCR kit (Takara, China), and the relative primers are presented in Table 2. The expression level of mRNAs was normalized to GAPDH (Sangon Biotech, Shanghai, China), respectively, as endogenous control and calculated using the 2^−ΔΔCt^ method.

### 2.10. Western Blot

Total cell protein was extracted using Qproteome Mammalian Protein Prep Kit (QIAGEN, Germany). The proteins were separated on 12% SDS-PAGE and transferred to PVDF membranes. The membranes were blocked in 3% skim milk for 1 h and incubated with *β*-actin (diluted 1 : 200, Santa Cruze, 47778, USA), IRAK1 (diluted 1 : 1000, Abcam, 238, UK), p-IRAK1 (diluted 1 : 500, Abcam, 218130, UK), NF-*κ*B p65 (diluted 1 : 1000, Cell Signaling Technology, 8242, USA), p-NF-*κ*B p65 (diluted 1 : 2000, Abcam, 178870, UK), TRAF6 (diluted 1 : 1000, Abcam, 33915, UK), IKB*α* (diluted 1 : 1000, Abcam 32518, UK), p-IKB*α* (diluted 1 : 5000, Abcam 133462, UK), and IKK*α* + IKK*β* (diluted 1 : 1000, Abcam 178870, UK) overnight at 4°C. Subsequently, the membranes were incubated with secondary antibody IRDye 800 goat anti-rabbit IgG (diluted 1 : 5000, LI-COR Bioscience, 926-32211, USA) or IRDye 680 goat anti-mouse IgG (diluted 1 : 5000, LI-COR Bioscience, 926-68070, USA) at room temperature for 1 h. Protein bands were detected using an Odyssey fluorescence scanner (LI-COR; Bioscience, Lincoln, NE, USA) and quantified using BIORAD Quantity One software (Bio-Rad, Hercules, CA, USA). Data were normalized against those of the corresponding *β*-actin values.

### 2.11. Statistical Analysis

All data were described as mean ± standard deviation from at least three independent experiments. Independent-sample *t*-test and one-way ANOVA test which were used in the present research were performed using Prism 6.0 (GraphPad Software, San Diego, CA, USA) and SPSS software (version19.0, IBM Corporation, Armonk, NY, USA). And, statistically significant changes were classified as significant while *P* < 0.05.

## 3. Results

### 3.1. Effects of JP on the Cell Proliferation of Peritoneal Macrophages in MRL/Lpr Lupus Mice

**Figure 1:**
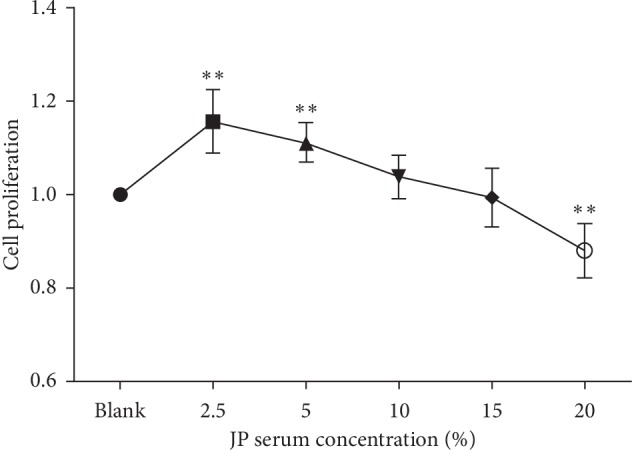
The effects of JP-treated rat serum on cell proliferation. Peritoneal macrophages of MRL/lpr lupus mice were seeded onto 96-wells plate and treated with various concentrations of JP-treated rat serum (0, 2.5, 5, 10, 15, or 20%) for 24 h Cell proliferation was assessed using CCK-8 assay. The values were expressed as the mean ± SD of three independent experiments. ^*∗*^*P* < 0.05, and ^*∗∗*^*P* < 0.01 versus blank cells.

We treated cells with concentration levels ranging from 2.5% to 20% of JP for 24 hours to investigate which level has the most significant effect on the proliferation of peritoneal macrophages in MRL/lpr lupus mice (Figure 1). CCK-8 assays were performed for optimal serum concentration selection and cell proliferation assessment. We observed a dose-related relationship between cell proliferation and serum concentration after JP treatment, but there was no significant alteration in cell viability. Simultaneously, 2.5% JP serum was the most effective concentration.

### 3.2. JP Can Suppress the IRAK1 Signaling Pathway Activated by LPS

**Figure 2:**
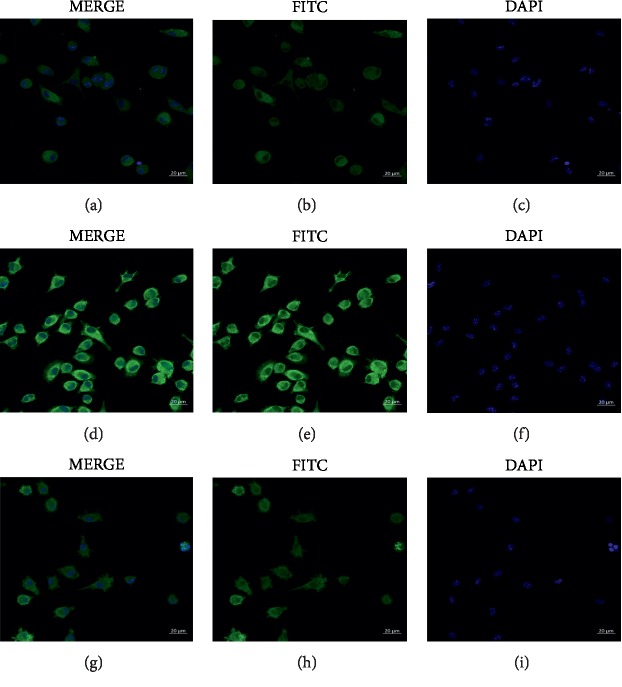
JP suppresses IRAK1 activation in LPS-stimulated peritoneal macrophages of MRL/lpr lupus mice. Cells were treated with LPS either alone or with JP (2.5%) for 24 h later, the cells were subjected by immunofluorescent assays using DAPI (blue) and FITC (green). Data are representative images (magnification, 40× for immunofluorescent staining) of individual groups from three separate experiments. A control group; B LPS group; C LPS plus JP group.

LPS is a major component of the cell wall of Gram-negative bacteria and plays an important role in the pathogenesis of SLE [24]. To further explain this phenomenon, we conducted an in vitro study. The cells were divided into three groups: control group, LPS group, and LPS plus JP group. The expression of IRAK1 in the peritoneal macrophages of MRL/lpr lupus mice was determined by immunofluorescence staining. As a result, expression of IRAK1 with FITC green fluorescence was observed (Figure 2). Compared with the control group, the expression of IRAK1 was significantly distributed in the LPS group, mainly in the cell membrane and cytoplasm. In the LPS plus JP group, the expression of IRAK1 was significantly decreased, mainly distributed on the cell membrane surface. The results showed that LPS could stimulate the expression of IRAK1 in macrophages, and the serum of JP could inhibit the expression of IRAK1 in macrophages.

**Figure 3:**
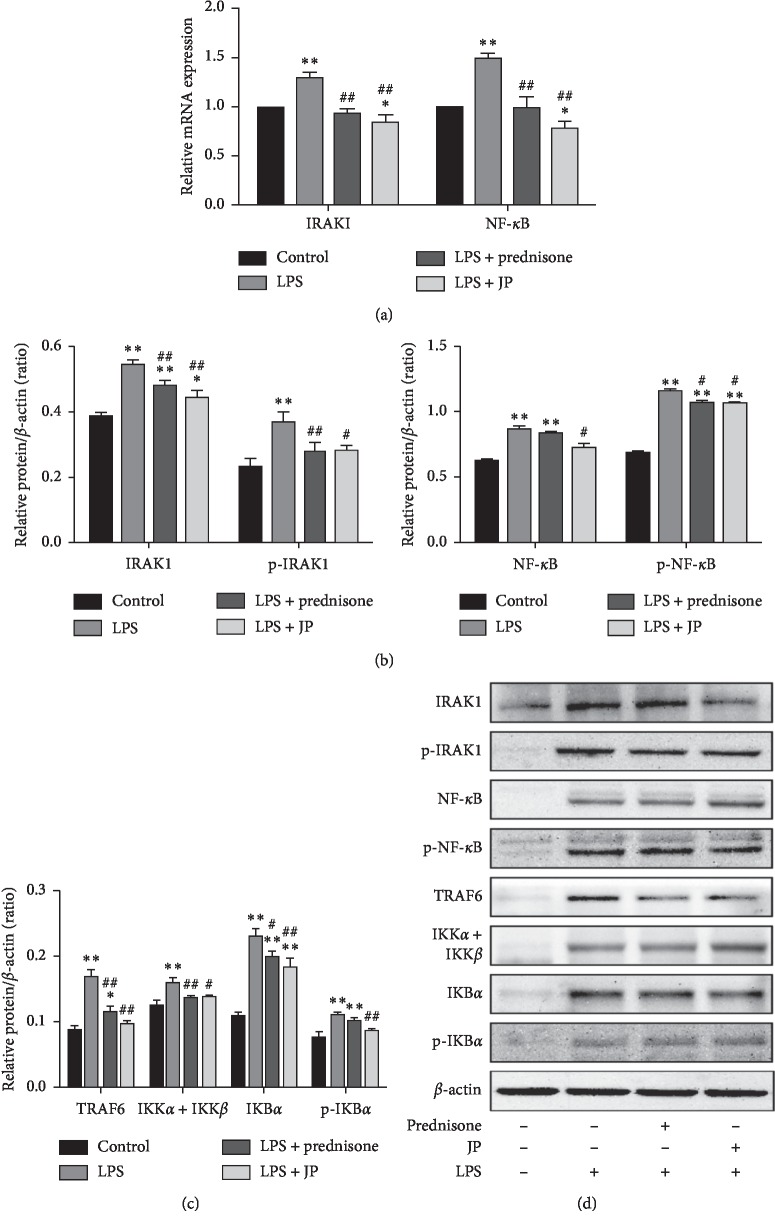
JP downregulates LPS-induced signaling pathway in peritoneal macrophages of MRL/lpr mice. (a) The expression alterations in IRAK1 and NF-*κ*B mRNA. (b) The expression alterations in phosphorylation and nonphosphorylation of IRAK1 and NF-*κ*B protein. (c) The protein expression alterations in TRAF6, IKB*α*, p-IKB*α*, and IKK*α* + IKK*β*. (d) The related protein bands. Values are expressed as mean ± SD of three independent experiments. ^*∗*^*P* < 0.05 and ^*∗∗*^*P* < 0.01 versus control cells; ^#^*P* < 0.05 and ^##^*P* < 0.01 versus LPS-treated cells.

Fluorescence quantitative PCR was performed on RNA isolated from the cells of the LPS group, LPS plus prednisone group, and LPS plus JP group and control group at 24 h. We first detected that mRNA expression of IRAK1 and downstream NF-*κ*B were significantly increased after LPS induction, but its expression decreased when JP intervened (*P* < 0.01) (Figure 3(a)). In addition, western blot was used to detect the inhibitory effect of JP on IRAK1-NF-*κ*B pathway. Consistent with Rt-PCR, we found LPS significantly activated the expression of IRAK1 and its downstream NF-*κ*B protein, at which time JP had a reducing effect (*P* < 0.05) (Figures 3(b) and 3(d)). Astonishingly, the proteins involved in the IRAK1 and NF-*κ*B pathways include TRAF6, IKB*α*, and p-IKB*α*, and IKK*α* + IKK*β* protein in the LPS group was significantly increased compared to the control group (*P* < 0.05) (Figures 3(c) and 3(d)). Furthermore, the results showed that however the reduction of IRAK1 and NF-*κ*B mRNA expression and the reduction of IRAK1, NF-*κ*B, TRAF6, IKB*α*, and p-IKB*α* protein expression in the JP group were not particularly significant compared with the prednisone group (*P* < 0.05). The trend of JP and prednisone acetate to reduce the IRAK1-NF-*κ*B signaling pathway was consistent. Altogether, these results demonstrate that JP can suppress the IRAK1 signaling pathway activated by LPS in peritoneal macrophages of MRL/lpr lupus mice.

### 3.3. JP Can Inhibit the Secretion of Inflammatory Cytokines in Peritoneal Macrophages of MRL/Lpr Lupus Mice

**Figure 4:**
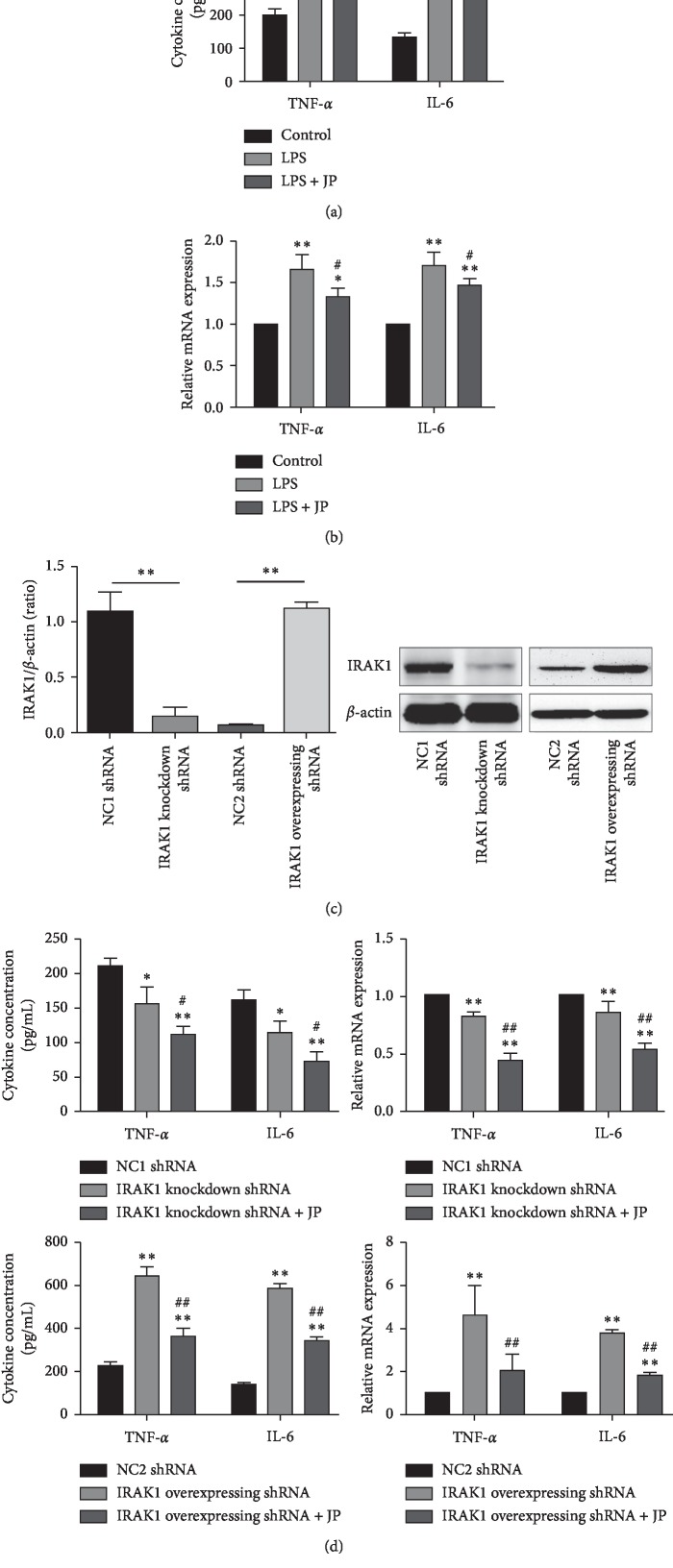
JP inhibits the secretion of inflammatory cytokines in peritoneal macrophages of MRL/lpr lupus mice. (a) The expression alterations in TNF-*α* and IL-6 mRNA. (b) Concentrations of TNF-*α* and IL-6 in the supernatant of peritoneal macrophages of MRL/lpr mice. (c) Transfection protein bands and transfection efficiency of IRAK1 shRNA in peritoneal macrophages of MRL/lpr mice. (d) After transfection and JP treatment, the alterations of inflammatory cytokine production and TNF-*α* and IL-6 mRNA in cells. Values are expressed as mean ± SD of three independent experiments. ^*∗*^*P* < 0.05 and ^*∗∗*^*P* < 0.01 versus vehicle control cells; ^#^*P* < 0.05 and ^##^*P* < 0.01 versus LPS or shRNA-treated cells.

To assess the inhibitory effects of JP, we treated LPS-contained peritoneal macrophages with or without JP for 24 h, and the production of TNF-*α* and IL-6 was analyzed using ELISA. Peritoneal macrophages containing LPS increased the inflammatory cytokine production compared with the control-treated cells. By contrast, JP significantly suppressed LPS-stimulated production of TNF-*α* and IL-6 (*P* < 0.05) (Figure 4(a)). We also analyzed the effects of JP on inflammatory cytokine mRNA in peritoneal macrophages. Consistent with the results of ELISAs, stimulation with LPS significantly increased the expression of TNF-*α* and IL-6 mRNA in cells. Treatment of the cells with JP significantly reduced the expression of TNF-*α* and IL-6 mRNA compared with LPS (*P* < 0.05) (Figure 4(b)).

To extend our in vitro study, IRAK1 knockdown and overexpressing shRNA were transfected into peritoneal macrophages, and no-knockdown as well as no-overexpress shRNA group were set as negative control. Furthermore, we assessed the expression of IRAK1 in these cells by western blot. The results indicate that the level of IRAK1 protein expression either decreased or increased by about 85% of control values in 72 h (*P* < 0.01) (Figure 4(c)). After transfection, the cells were divided into six groups: IRAK1 knockdown/overexpressing shRNA group and negative control shRNA group (NC1 shRNA/NC2 shRNA) were treated with complete culture medium containing JP-free rat serum, whereas IRAK1 knockdown/overexpressing shRNA plus JP group were treated with complete culture medium containing JP rat serum; all groups were incubated in culture medium for 24 h. As a result, compared to the NC1 shRNA group, the IRAK1 knockdown shRNA group significantly decreased the expression of inflammatory cytokines TNF-*α*, IL-6 and TNF-*α*, IL-6 mRNA (*P* < 0.01). After JP intervention, the expression of inflammatory cytokines TNF-*α*, IL-6 and TNF-*α*, IL-6 mRNA was decreased (*P* < 0.05) (Figure 4(d)). At the same time, IRAK1 and its downstream pathyway were activated after IRAK1 being overexpressed, JP intervention significantly decreased the expression of inflammatory cytokines TNF-*α*, IL-6 and TNF-*α*, IL-6 mRNA (*P* < 0.05) (Figure 4(d)). The results suggested that JP inhibited the secretion of inflammatory cytokines in peritoneal macrophages of MRL/lpr lupus mice through the IRAK1 pathway.

### 3.4. JP Inhibits the Activation of Peritoneal Macrophages in MRL/Lpr Lupus Mice by Downregulating IRAK1 and NF-*κ*B Signaling Pathway

**Figure 5:**
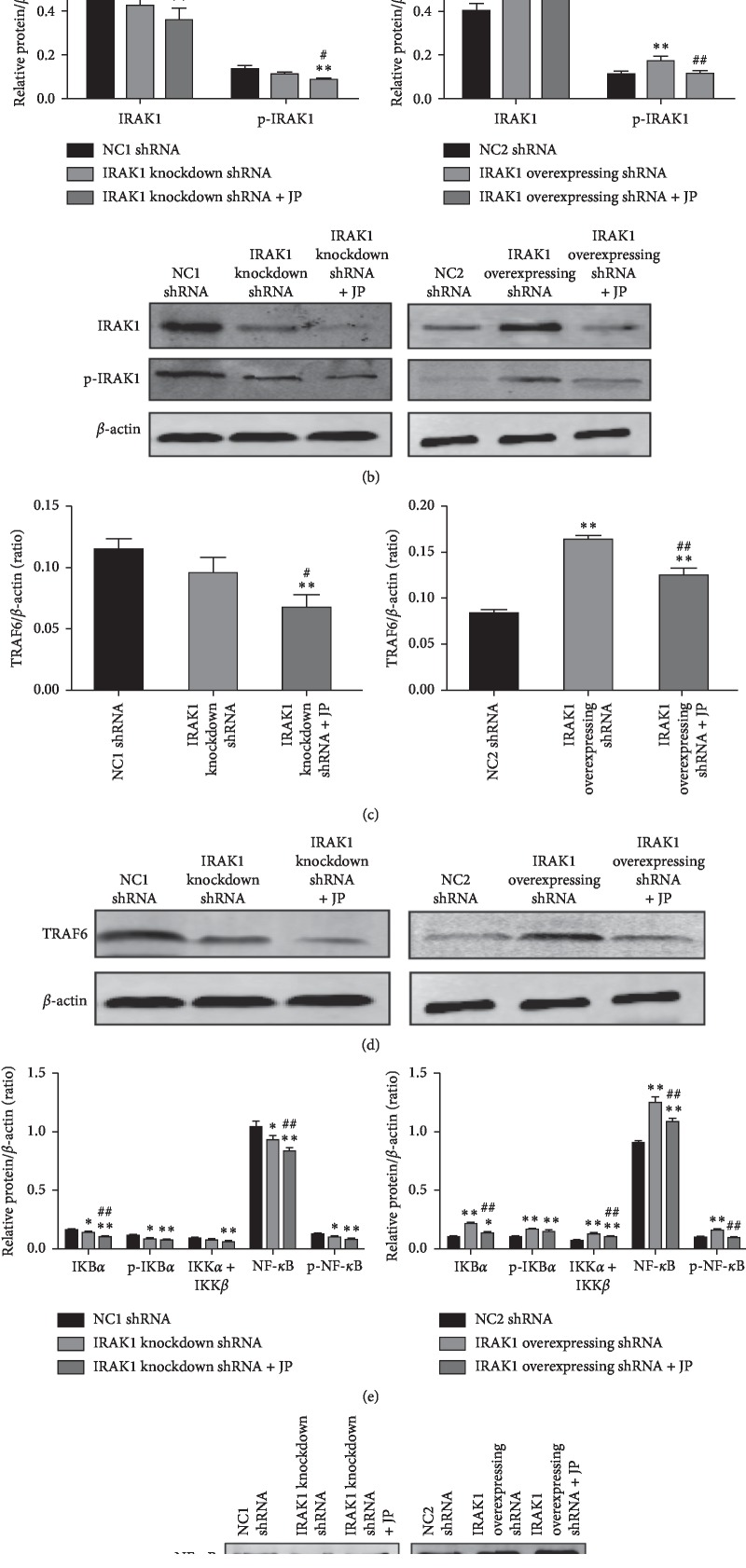
JP inhibits the activation of peritoneal macrophages in MRL/lpr lupus mice by downregulating IRAK1 and NF-*κ*B signaling pathway. (a) After transfection and JP treatment, the expression alterations in IRAK1 and NF-*κ*B mRNA. (b) First of all, JP could inhibit the expression of IRAK1 protein. (c) Next, after the JP intervention, the level of TRAF6 protein was inhibited. (d) Protein bands of each group of TRAF6. (e) Finally, the expression alterations in IKB*α*, p-IKB*α*, IKK*α* + IKK*β*, NF-*κ*B, and p-NF-*κ*B protein after transfection and JP treatment. (f) Protein bands of each group of the related antibodies. Values are expressed as mean ± SD of three independent experiments. ^*∗*^*P* < 0.05 and ^*∗∗*^*P* < 0.01 versus vehicle control cells; ^#^*P* < 0.05 and ^##^*P* < 0.01 versus shRNA-treated cells.

IRAK1 plays an important role in the progression of SLE [25]. Therefore, we aimed to determine if JP inhibits the activation of peritoneal macrophages in MRL/lpr lupus mice by downregulating IRAK1 and NF-*κ*B signaling pathway. We then performed Rt-PCR and western blot for mRNA and protein in the above shRNA groups; as expected, the results showed that JP serum could inhibit the expression of IRAK1 mRNA and protein (*P* < 0.05) (Figures 5(a) and 5(b)).

Activation of IRAK1 and TRAF6 was interacted to promote NF-*κ*B activation, and the results showed that TRAF6 expression increased in macrophages after IRAK1 overexpression shRNA transfection (*P* < 0.05). After JP intervention, the level of TRAF6 protein was inhibited (*P* < 0.01) (Figures 5(c) and 5(d)). It has been confirmed that the abnormal expression of IRAK1 could activate the downstream NF-*κ*B pathway, and therefore the inhibition of NF-*κ*B pathway by JP is detected by Rt-PCR (Figure 5(a)). The results showed that IKB*α*, p-IKB*α*, IKK*α* + IKK*β*, NF-*κ*B, and p-NF-*κ*B protein and phosphorylated protein were increased in the IRAK1 overexpressing shRNA group, compared with the NC2 shRNA group (*P* < 0.05). However, these trends are inhibited by JP serum (Figures 5(e) and 5(f)).

At the same time, in the IRAK1 knockdown shRNA group, the interference of IRAK1 shRNA significantly decreased the expression of IRAK1-NF-*κ*B signaling pathway (*P* < 0.05), and the IRAK1-NF-*κ*B signaling pathway was further reduced after the intervention of JP (*P* < 0.05) (Figures 5(a)–5(f)). As expected, IRAK1 overexpressing shRNA significantly upregulated the expression of the IRAK1-NF-*κ*B signaling pathway (*P* < 0.05). We found that the IRAK1-NF-*κ*B signaling pathway was abnormally activated, and the expression of the IRAK1-NF-*κ*B signaling pathway was significantly decreased after adding JP (*P* < 0.05). Altogether, JP can inhibit the activation of peritoneal macrophages in MRL/lpr lupus mice by downregulating the phosphorylation and nonphosphorylation of IRAK1 and NF-*κ*B signaling pathway.

## 4. Discussion

Macrophages, which can effectively phagocytose pathogens, are the first line of defense against foreign antigens. Thereby, it exerts antigen-presenting effects and defense, inflammatory regulation, and immune-induced functions. Studies have shown that abnormalities in macrophage function could break the body's immune homeostasis and induce autoimmune diseases [17–19]. Toll-like receptors (TLRs) are important members of pattern recognition receptors expressed by macrophages, and macrophages mainly express TLR2/4. The expression of TLR4, involved in a variety of inflammatory responses, is a major source of inflammatory cytokines in the body, and thus it plays an important role in the pathogenesis of different diseases [26]. Meanwhile, the surface of macrophages has TLR4 receptors that can be stimulated by LPS and are also the main receptors for the identification of LPS [27]. LPS is the main component of the cell wall of Gram-negative bacteria and plays an important role in the pathogenesis of SLE [24]. Studies have shown that compared with healthy people, macrophage-related surface molecules, immune regulation, and phagocytosis in SLE patients are abnormal, suggesting that macrophage abnormalities are associated with the pathogenesis of SLE and that targeting of macrophages may be a new strategy to improve the treatment of SLE [21]. Therefore, in this experiment, we selected primary cells such as MRL/lpr lupus mouse peritoneal macrophages as the research object and explored the mechanism of action between SLE and IRAK1 signaling pathway from the cellular level.

Treatment-related side effects are common in SLE patients; hence, it is important to discover new therapeutic agents aiming at the novel molecular targets for SLE progression. This study indicated that IRAK1 might be a new target for SLE intervention. Firstly, we found that IRAK1 expression and NF-*κ*B expression were significantly upregulated in peritoneal macrophages from lupus-prone MRL/lpr mice. These results support the notion that IRAK1 is a risk factor for SLE and that aberrant activation of NF-*κ*B signaling is associated with the pathogenesis of human SLE [28, 29]. Besides, miRNA inhibition of IRAK1 expression promotes DC apoptosis, suggesting that IRAK1 positively regulates DC survival and enhances inflammation [30]. Thus, IRAK1 may contribute to the pathogenesis of SLE and other inflammatory diseases by enhancing NF-*κ*B signaling, proinflammatory cytokine production, and DC survival.

Recent studies have shown that inhibition of IRAK1 could ameliorate IKB*α* phosphorylation, which in turn inhibits NF-*κ*B signaling and downstream proinflammatory cytokine production in immunocompetent cells, reducing lupus-related renal damage. NF-*κ*Bp65 is a widely expressed nuclear transcription factor that plays an important role in the regulation of various inflammatory mediators, usually in the cytoplasm. Phosphorylation of IKB*α* allows NF-*κ*Bp65 to translocate to the nucleus and induce transcription [31]. Moreover, recent studies have determined that IRAK1 was a risk factor for the development of SLE [32]. IRAK1 inhibitors can attenuate NF-*κ*B signaling in spleen mononuclear cells of lupus-prone lpr mice and SLE patients with PBMCs, while it reduces proinflammatory cytokine production in mice. Hence, the inhibition of IRAK1 activity may be helpful in discovering new therapies for SLE and other inflammatory diseases. The JP in this experiment has an effect similar to the IRAK1 inhibitor.

TCM is considered a complementary and alternative medicine that emphasizes the “individualized” diagnosis and treatment of patients [33]. Our previous studies have shown that JP could slow down or prevent disease progression without significant toxic side effects [5, 9]. Thus, the mechanism of action of JP for the treatment of SLE can be understood [34]. Therefore, in this study, we selected primary peritoneal macrophages induced by LPS and transfection with shRNA lentiviral and then analyzed JP-to-IRAK1 signaling pathway intervention from changes in downstream molecules TRAF6, IKB*α*, IKK, and NF-*κ*B.

In this study, the results showed that the expression of NF-*κ*B and inflammatory factors TNF-*α* and IL-6 in the inflammatory signaling pathway was significantly increased after LPS stimulation of peritoneal macrophages in MRL/lpr mice. This indicates that the specific binding of LPS to Toll-like receptors, particularly TLR4, aberrantly activates the IRAK1 signaling pathway, thereby initiating the transcription of immune-related genes and inducing various inflammatory responses. Furthermore, in this experiment, IRAK1 was overexpressed by shRNA lentivirus transfection and then acted on cells. As a result, the expression of the IRAK1 signaling pathway was significantly increased after overexpression, and the expression of downstream molecules increased. At the same time, this study also verified the expression of IRAK1 in peritoneal macrophages of MRL/lpr mice by gene knockdown. The results showed that the protein and mRNA of IRAK1 more obviously changed after JP acted on peritoneal macrophages of lupus mice, to be exact, is to suppress. The change of p-IRAK1 protein further indicates that the decrease of phosphorylation level is more obvious. More interestingly, the expression of TRAF6, IKB*α*, IKK, and NF-*κ*B in the downstream molecules of IRAK1 decreased, and the phosphorylation level decreased significantly. All of the above indicate that JP can inhibit IRAK1 and downstream phosphorylation levels in macrophages from lupus mice; thus, it plays a vital role in the pathological process of SLE. However, the levels of inflammatory cytokines TNF-*α* and IL-6 decreased after JP intervention in this experiment, which indicates the effective inhibition of JP in the IRAK1-NF-*κ*B inflammatory signaling pathway. Further validation of our hypothesis is that JP helps to inhibit the onset of SLE by inhibiting the expression of the IRAK1 signaling pathway.

IRAK1 may be an important factor and a new therapeutic target for the pathogenesis of SLE or other proinflammatory diseases. Our study shows that JP has a protective effect on SLE and inhibits the secretion of proinflammatory cytokines by IRAK1-mediated NF-*κ*B signaling. Taken together, JP may play an important role in SLE intervention by inhibiting the potential target of IRAK1. The results of this study provide a new clinical application of IRAK1 for the treatment of SLE, which lays the foundation for the promotion of JP and more traditional Chinese medicine as well as the further research of new IRAK1 inhibitors.

## 5. Conclusion

We demonstrated that Jieduquyuziyin prescription- (JP-) treated rat serum could significantly reduce the levels of proinflammatory cytokines TNF-*α* and IL-6, IRAK1, NF-*κ*B, TNF-*α* and IL-6 mRNA expression, and expression of IRAK1, p-IRAK1, TRAF6, IKB*α*, p-IKB*α*, IKK + IKK, NF-*κ*B, and p-NF-*κ*B proteins in IRAK1-NF-*κ*B signaling pathway activated by peritoneal macrophages of MRL/lpr mice, thereby inhibiting IRAK1 signaling and regulating immune function effect. Furthermore, JP may inhibit the activation of peritoneal macrophages in MRL/lpr mice by downregulating the IRAK1-NF-*κ*B signaling pathway, which may be helpful in studying the mechanisms of SLE and other proinflammatory diseases and in finding new therapeutic targets. Meanwhile, these findings have inspired us that TCM can be considered as an effective supplement and alternative medicine for SLE inflammation treatment, which also encourages further research into this treatment.

## Figures and Tables

**Table 1:** The compositions of Jieduquyuziyin (JP) formula.

Chinese name	Botanical name	Latin name	Parts used	Proportion (%)
Gan Di Huang	*Rehmannia glutinosa* Libosch.	*Rehmanniae radix*	Root tuber	12.8
Zhi Bie Jia	*Trionyx sinensis* Wiegmann	*Trionycis carapax*	Tergum	10.3
Qing Hao	*Artemisia annua* L.	*Artemisiae annuae* herba	Herb	10.3
Bai Hua She She Cao	*Hedyotis diffusa* Willd.	*Herba hedyotidis*	Herb	12.8
Chi Shao	*Paeonia veitchii* Lynch	*Paeoniaeradix rubra*	Root	10.3
Ji Xue Cao	*Centella asiatica* (L.) Urb.	*Centellae herba*	Herb	12.8
Dan Pi	*Paeonia suffruticosa* Andr.	*Moutan cortex*	Root	10.3
Fo Shou	*Citrus medica* L.	*Citri sarcodactylis* fructus	Fruit	7.7
Sheng Ma	*Cimicifuga foetida* L.	*Cimicifugae rhizoma*	Rhizome	7.7
Sheng Gan Cao	*Glycyrrhiza uralensis* (Fisch.)	*Glycyrrhiza uralensis* Fisch	Root	5.0

**Table 2:** List of primer sequences for RT-PCR.

Primer name	Sequence
IRAK1 forward	5′-GGTCCCTGTCTCTTCCCTTC-3′
IRAK1 reverse	5′-GAGGAAGGAATTCAGCCTTTG-3′
NF-*κ*B forward	5′-GCCGTGGAGTACGACAA-3′
NF-*κ*B reverse	5′-CGGTTTCCCATTTAGTATGT-3′
TNF-*α* forward	5′-ACCAGACACCTCAGGGCTAA-3′
TNF-*α* reverse	5′-TGTTGGGGAGAAGGAGAATG-3′
IL-6 forward	5′-AGCCAGAGTCCTTCAGAGAGATAC-3′
IL-6 reverse	5′-AATTGGATGGTCTTGGTCCTTAGC-3′

## Data Availability

The data used to support the findings of this study are available from the corresponding author upon request.
